# IM-TORNADO: A Tool for Comparison of 16S Reads from Paired-End Libraries

**DOI:** 10.1371/journal.pone.0114804

**Published:** 2014-12-15

**Authors:** Patricio Jeraldo, Krishna Kalari, Xianfeng Chen, Jaysheel Bhavsar, Ashutosh Mangalam, Bryan White, Heidi Nelson, Jean-Pierre Kocher, Nicholas Chia

**Affiliations:** 1 Department of Surgery, Mayo Clinic, Rochester, Minnesota, United States of America; 2 Institute for Genomic Biology, University of Illinois at Urbana-Champaign, Urbana, Illinois, United States of America; 3 Department of Health Sciences Research, Mayo Clinic, Rochester, Minnesota, United States of America; 4 Department of Immunology, Mayo Clinic, Rochester, Minnesota, United States of America; 5 Department of Animal Sciences, University of Illinois at Urbana-Champaign, Urbana, Illinois, United States of America; 6 Department of Physiology and Biomedical Engineering, Mayo Clinic College of Medicine, Rochester, Minnesota, United States of America; Belgian Nuclear Research Centre SCK•CEN, Belgium

## Abstract

**Motivation:**

16S rDNA hypervariable tag sequencing has become the *de facto* method for accessing microbial diversity. Illumina paired-end sequencing, which produces two separate reads for each DNA fragment, has become the platform of choice for this application. However, when the two reads do not overlap, existing computational pipelines analyze data from read separately and underutilize the information contained in the paired-end reads.

**Results:**

We created a workflow known as Illinois Mayo Taxon Organization from RNA Dataset Operations (IM-TORNADO) for processing non-overlapping reads while retaining maximal information content. Using synthetic mock datasets, we show that the use of both reads produced answers with greater correlation to those from full length 16S rDNA when looking at taxonomy, phylogeny, and beta-diversity.

**Availability and Implementation:**

IM-TORNADO is freely available at http://sourceforge.net/projects/imtornado and produces BIOM format output for cross compatibility with other pipelines such as QIIME, mothur, and phyloseq.

## Introduction

The advent of high-throughput 16S rDNA microbial population sequencing has revolutionized our ability to carry out culture independent surveys [1] and paved the way to understanding the microbiome from environments ranging from the practical [2], [3] to the exotic [4], [5]. The need for bioinformatics has grown hand-in-hand with the increasing depth and complexities of microbiome analyses [6], [7]. The importance of being able to organize such analyses is epitomized by the popularity of microbiome analysis pipelines such as QIIME [8] and mothur [9], which collectively to date have more than 3000 citations.

Historically, most microbiome analyses were carried out using large amplicons (>500 bp long) sequenced on the 454 pyrosequencing platform. Recently, due to its higher throughput and multiplexing capability, Illumina sequencers have become the preferred platform for microbiome analyses, despite the shorter reads produced (100 to 300 bp long) [10]–[12]. The Illumina platform is currently used by the microbiome community following two preferred approaches. The first approach consists in designing small amplicons, to force the reads from the same amplicon ends to overlap. These reads can then be assembled into a single read longer than the 250 bp reads, thereby increasing their specificity. The second approach focuses on sequencing the 250 bp ends of the large amplicons originally crafted for the 454 platform. These regions have been previously studied [13], [14] and optimized for capturing phylogenetic or taxonomic information. However, this can lead to reads that do not overlap when sequenced on the Illumina platform [15]–[18].

The use of non-overlapping paired reads requires a large amount of tracking to maintain the proper linkages while preserving the necessary efficiency expected of 16S rDNA analyses, which typically take advantage of redundancy in the datasets. While cases have been explored previously [16], existing analytical pipelines are only built for analyzing single reads and do not track the linkages between the two non-overlapping reads produced by Illumina, and such tracking can create a “potentially more complicated downstream pipeline” [19]. This can create a conundrum for the researcher who may have to choose drawing conclusions based on a single read [20], or altering their primer design to allow for overlapping reads [11]. These former uses less than the maximum information available and are suboptimal in the accuracy of the analyses they provide while the bioinformatics for the latter has already been solved elsewhere by assembling the paired-end reads [21], [22].

Here, we present an integrated workflow, known as the Illinois-Mayo Taxon Organization from RNA Dataset Operations (IM-TORNADO), for carrying out common microbiome analyses leveraging the information of the paired reads provided by the Illumina sequencers to relate reads belonging to the same amplicon, making the use of these non-overlapping reads accessible to a broader base of users. We use our pipeline to compare the accuracy of results obtained from paired reads versus single reads. We find that with regards to three common operations–making multiple alignment, inferring taxonomy, and phylogeny– utilizing the information contained in both reads leads to better performance than analysis of any single read. Where possible, we build upon existing tools and link to formats for further processing other popular pipelines [8], [9], [23] in order to augment the growing community of computational tools for microbiome analyses. In this article, we will first outline the general methodology of IM-TORNADO and compare the performance of with IM-TORNADO when using paired reads versus either read individually. We then describe, in detail, the methods employed at every step. Finally, we discuss the significance of IM-TORNADO's improved performance using paired reads and its impact on future experimental designs.

## Approach

Paired-end sequencing produces 2 reads, read 1 (R1) and read 2 (R2), each from opposite ends of a DNA fragment. In the case of 16S rDNA hypervariable tag sequencing, these regions are selected by primers targeted to specific regions. We approach the case where R1 and R2 do not overlap and the sequencing of the amplified DNA fragment is incomplete due to the gap between the two reads. Algorithms for taxonomy, multiple alignment, and phylogeny that do not take into account this gap cannot be employed naively and require an organizational framework to properly manage the link between each R1 and R2 read pair.

In this section, we describe results of an analysis pipeline for preparing non-overlapping reads for analysis as a whole unit, without sacrificing one of the reads in the pair. Our approach, IM-TORNADO, employs a number of smaller scripts that uniformize and merge paired-reads for analysis by widely-used analytical tools, guaranteeing reasonable comparability with existing studies. We test our approach using a synthetic mock dataset that simulates 16S rDNA hypervariable tag sequencing of V3–V5 and V6–V9 regions of 16S rDNA and show that IM-TORNADO allows us to extract meaningful improvements in accuracy from non-overlapping paired read analyses in comparison to single-end read analyses. Details of our methodology are provided in the “Methods for Pipeline Implementation” section, and information about operational taxonomic units in these synthetic mock communities is discussed in the S1 Text and S2 Table.

### Taxonomy

Determining taxonomy relies on profiling characteristics or alignments of sequences in a reference database. 16S rDNA-based classification tools such as Ribosomal Database Project (RDP) taxonomy classifier use 16S rDNA databases such as Greengenes [24]. Algorithms for determining taxonomy expect an ungapped sequence input, adding a layer of difficulty when utilizing non-overlapping paired-end reads. We joined the paired-end reads using an ambiguous nucleotide character before the data is input into the classifier in order to avoid misinterpretation of the data while still retaining the information from both reads.

Table 1 shows the overall accuracy of taxonomic calls using paired, R1, R2, and full length 16S rDNA. Accuracy was determined by comparison to the original sequence in the synthetic mock dataset, whose taxonomy is pre-determined by the reference database, in this case, Greengenes 13_–_5. Paired reads perform better than either single end read, and paired reads generally outperform R1 by about the same amount by which full length 16S rDNA outperforms the paired reads. In the S1 Text we also discuss the effect of read quality errors and error-trimming on the accuracy of the taxonomy assignment (see also S1 Table).

**Table 1:** Taxonomy comparison.

Library type	Domain	Phylum	Class	Order	Family	Genus	Species
Paired	100.0	99.95	99.91	99.59	98.25	94.32	90.78
R1	100.0	99.91	99.83	99.18	97.41	92.12	87.60
R2	100.0	99.89	99.76	99.10	97.14	87.38	80.47
Full length	100.0	99.99	99.97	99.68	99.88	96.30	94.57

Comparison of accuracy from taxonomy calls using paired, read 1 (R1), read 2 (R2), and full length 16S rDNA. Analyses were carried out using the Ribosomal Database Project taxonomy classifier with the complete Greengenes database. Using Paired read analysis provides more accurate taxonomic classification than either R1 or R2 alone across all taxonomic levels. Full length 16S rDNA was used for comparison purposes.

### Multiple Sequence Alignment

Before making a comparison of phylogenies, we must make sure we make a sensible choice for the tool used to create the sequence alignment used for the phylogenies. In the case of high-throughput 16S rDNA sequencing, specialized tools exist to guarantee a good alignment (based on the extensive knowledge of ribosomal RNA) while performing a very fast multiple sequence alignment. Two tools stand out for this task. The Nearest Alignment Space Termination (NAST) algorithm [25] uses a template of pre-aligned sequences which are used to find the closest template reads matching the query read, align to the template, and carefully introduce misalignments to make sure the query matches the template structure. The other tool, Infernal [26] is a more general-purpose RNA toolkit which contains a module which aligns queries to a reference secondary structure. In particular, a set of 16S reads is used by the RDP Pipeline to create a secondary structure model using Infernal, and used to align query reads.

**Figure 1:**
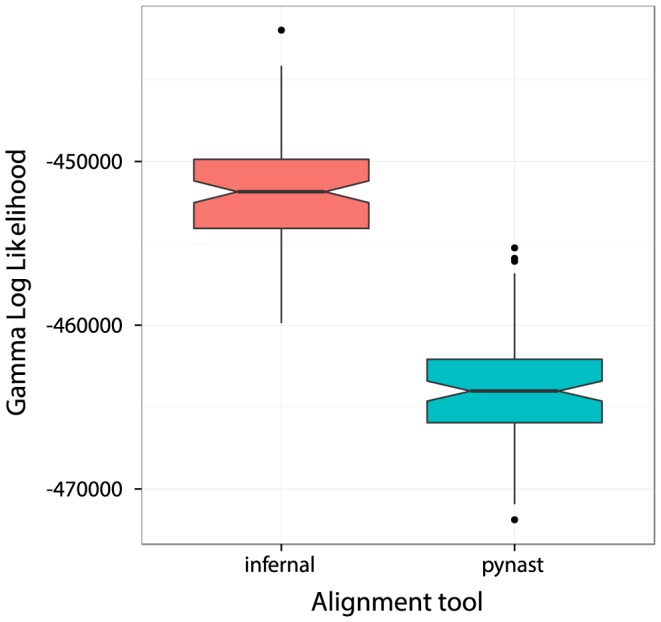
Comparison of alignment tools. Plot of Gamma Log Likelihoods of 100 trees created from paired reads selected from the Greengenes 13_–_5 database, and aligned using PyNAST and Infernal version 1.1. Likelihoods in the trees created using Infernal are significantly better than the trees created with PyNAST (*p*<0.0001, Wilcoxon signed ranked test), strongly suggesting that Infernal produces better quality alignments than PyNAST for the same input reads.

In Fig. 1, we show the result of comparing 100 phylogenetic trees (see Methods), created from the same reads randomly chosen from the Greengenes 13_–_5 database, but using different sequence aligners. We compared PyNAST [27] as bundled with QIIME version 1.8.0 (relaxing the minimum alignment identity threshold down to 50%, to ensure all queries are processed), and Infernal version 1.1 [28]. We compare the maximum likelihood trees created using FastTree version 2.7.1 [29] by examining the Gamma Log Likelihoods reported for each tree. The trees created using Infernal have significantly higher log likelihoods (*p*<0.0001, Wilcoxon signed ranked test) than the trees created using PyNAST, meaning the multiple sequence alignments are of higher quality. This is in agreement with previous results comparing these two algorithms [30].

### Phylogeny

Phylogenetic trees are often used as part of the metric of choice [31] when comparing organismal differences between communities. Using the multiple alignment scheme from Section “Multiple Sequence Alignment” to faithfully incorporate both non-overlapping reads R1R2, we are able to obtain more accurate phylogenies than using either R1 or R2.

**Figure 2:**
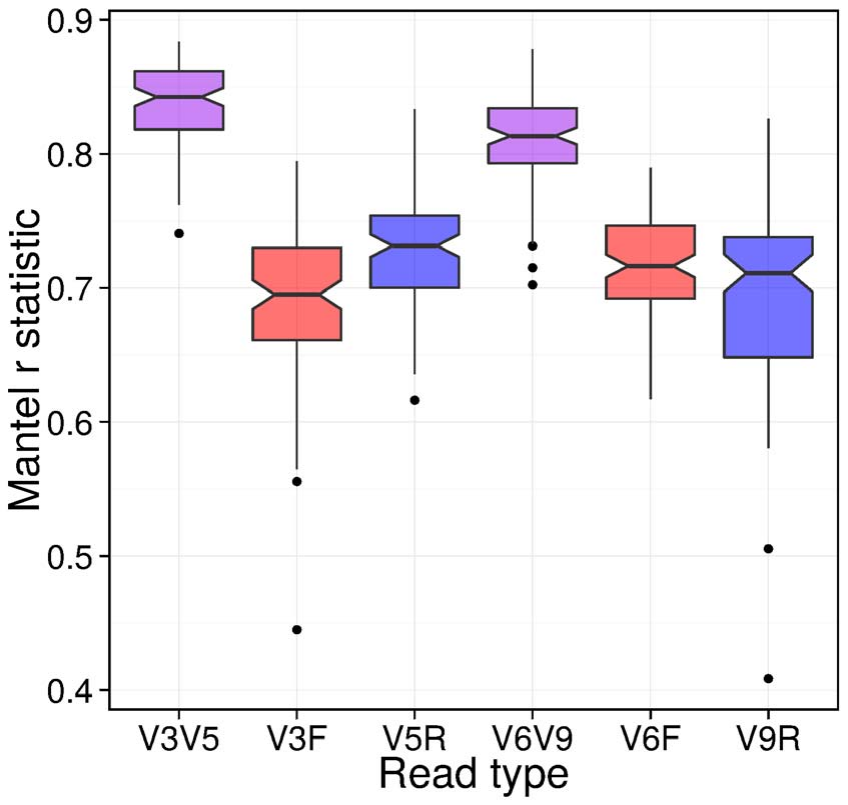
Comparison of phylogenetic trees between libraries. Plot of a Mantel correlation test comparing cophenetic distance matrices calculated from phylogenetic trees created using paired, R1 and R2 (for both the V3–V5 and V6–V9 primer pairs) versus the distance matrix created from the corresponding full-length 16S trees. A higher correlation value means the trees are more closely related to the full-length trees. Here, the paired trees are significantly closer to the full-length trees than the R1 and R2 trees (*p*<0.0001, Wilcoxon signed ranked test, using 100 synthetic mock communities), strongly suggesting that combining the use of paired reads leads to phylogenies closer to what is obtained from full-length reads, even when the chosen primers create non-overlapping reads.

We examine the quality of a given tree generated from paired-end sequencing data by looking at how well it agrees with a tree generated using the full length 16S rDNA from our synthetic mock dataset. Greater correlation between the branch distances indicates more similarity between the trees. A tree that agrees well with its full length counterpart captures the relationships between samples. Fig. 2 shows the results from 100 comparisons between full length 16S rDNA phylogeny and its short read counterparts generated using paired reads, R1, or R2. As shown, paired read analysis preserves significantly more of the phylogenetic tree structure than either R1 or R2 alone.

### *β*-diversity

Similarities and differences between microbial communities, or *β*-diversity, are often calculated from a phylogenetic tree. Here, we investigate the effect of using paired reads R1R2 versus single reads R1 or R2 on the fidelity of *β*-diversity.

**Figure 3:**
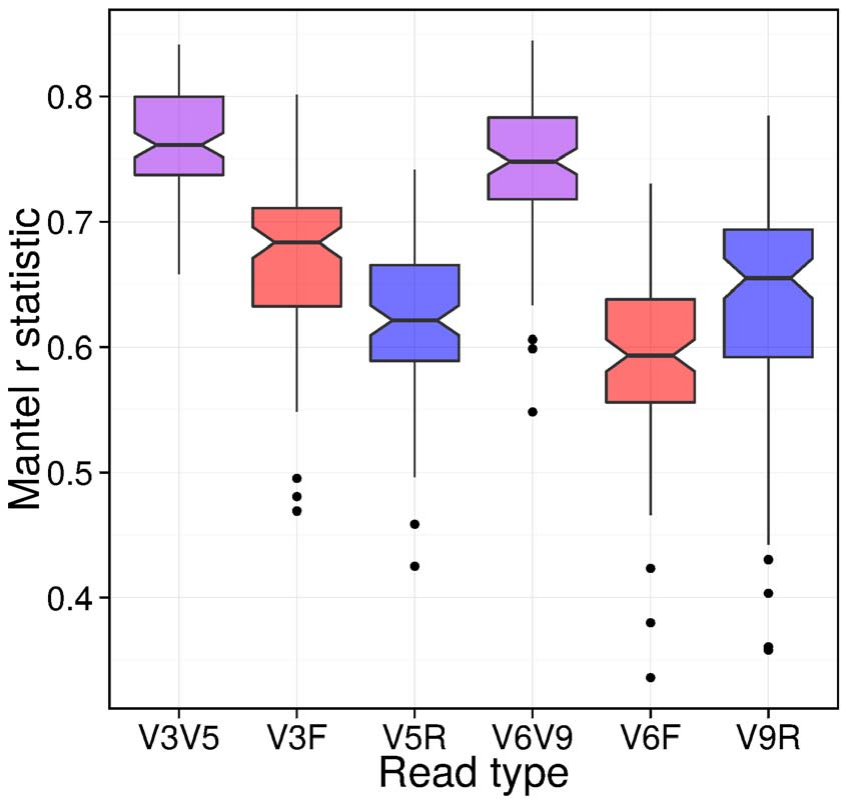
Comparison of *β*-diversity between libraries. Plot of a Mantel correlation test comparing unweighted UniFrac distance matrices created using synthetic mock communities from paired, R1 and R2 reads (for both the V3–V5 and V6–V9 pairs) versus the distance matrix created from the corresponding full-length 16S synthetic mock communities. A higher correlation value means the distance matrices, and hence their *β*-diversity, are more closely related to the full-length communities. Here, the communities from paired reads are significantly closer to the full-length communities than the R1 and R2 communities (*p*<0.0001, Wilcoxon signed ranked test, using 100 synthetic mock communities), strongly suggesting that combining the use of paired reads leads to results closer to what is obtained from full-length reads, even when the chosen primers create non-overlapping reads.

We examine the effect of using paired versus single-end reads in determining *β*-diversity between 100 randomly constructed communities. In order to assess accuracy, we use the corresponding full length 16S rDNA sequences from the reference database used to construct the synthetic mock dataset. Fig. 3 shows a comparison between the *β*-diversity distance matrices generated from paired, R1 and R2 reads. Paired reads retain a significantly greater amount of information than either single-end read alone.

### Use Case

The purpose of this use case test was to create a synthetic dataset that contains similar diversity and relatedness of organisms that might be found in a real dataset. For our example case, we chose to mimic the characteristics of a stool dataset (included with the validation datasets) by using closed reference mapping to full-length 16S rDNA from Greengenes 13_–_5 (see Methods). These full-length 16S rDNA reference reads then allowed us to the reproduce multiple artificial datasets using different primer regions what we could then compare against full length 16S rDNA reads. We then tested the different read libraries by computing their respective unweighted UniFrac matrices and compared the results of full-length to paired, R1 and R2 libraries using a Mantel correlation test. In Table 2, we show the results of the Mantel correlation test. We see that the paired reads have a higher correlation to the full-length reads than the single reads. This is concordant with the results shown in Fig. 3, which shows we expect to capture the same improvements when using real data.

**Table 2:** Library comparison in a realistic sample.

Library type	Mantel *r* statistic
Paired	0.931
R1	0.910
R2	0.813

Mantel *r* statistic comparing the unweighted UniFrac matrices for different short-read 16S libraries against a full-length 16S library, based on a real paired-end libary sequenced from stool samples. The paired reads have a higher correlation to the full-length library than any of the other single read libraries.

## Methods for Pipeline Implementation

**Figure 4:**
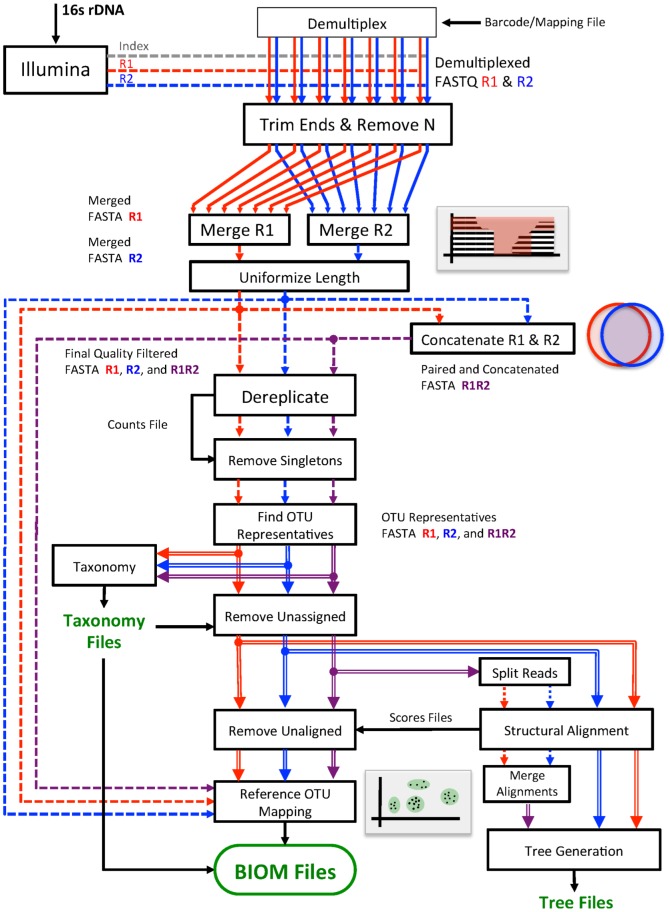
IM-TORNADO pipeline workflow. Schematic of the IM-TORNADO pipeline workflow.

In the following, we describe the steps performed by the IM-TORNADO pipeline to clean, merge, track, pick OTU, infer taxonomy and calculate phylogeny for a project. A graphical outline of the pipeline is presented in Fig. 4

### Input Data

The input for the IM-TORNADO pipeline is a set of demultiplexed fastq-formatted files. A metadata file describes the samples and other data the end user wants to be included in the output BIOM-formatted file.

### Quality Filtering

Demultiplexed sequence files are subject to quality filtering using Trimmomatic [32] version 0.30, with a hard cutoff of PHRED score Q3 for 5′ and 3′ ends of the reads (parameters LEADING: 3 and TRAILING: 3), trimming of the 3′ end with a moving average score of Q15, with a window size of 4 bases (parameter SLIDINGWINDOW: 4:15), and removing any remaining reads shorter than 75% [10] of the original read length (for example, parameter MINLEN: 112 for 150 bp long reads, MINLEN: 187 for 250 bp long reads or MINLEN: 225 for 300 bp long reads). Reads with any ambiguous base calls are discarded.

### Merging of Reads, Length Trimming and Concatenation

Surviving read pairs are grouped into two files, one for “read 1” (R1) and one for “read 2” (R2) sequences. We keep track of the sample of origin for each read, for later use in writing out the final OTU observation table. We trim the read lengths down to a specified cutoff. For R1, we keep the whole read; for R2, we trim down to around 88% of the maximum read length (e.g. 200 bp long for 250 bp long reads), given the overall lower sequencing quality of read 2. This uniformizes the length of both reads to satisfy the global alignabiltiy requirement of UPARSE [33]. At this stage, we keep all reads shorter than this cutoff. A third file is created by concatenating the matching trimmed reads from both reads 1 and read 2. Only reads with matching pairs in both read files are considered. This file will be used for further processing. Finally, a fourth file is created by stitching matching reads with an ambiguous nucleotide character “N” between read 1 and read 2. This file is to be used for taxonomy assignment, where a k-mer based approach will ignore k-mers containing ambiguous bases during the classification steps.

### Find OTU Representatives

The reads from the “Merging of Reads, Length Trimming and Concatenation” section are dereplicated, building clusters of reads with 100% similarity using mothur, and annotated with cluster size. To ensure the use of high quality reads when finding the OTU representatives, reads shorter than the cutoff length are discarded, as well as singleton reads. Reads are sorted by cluster size and processed using USEARCH to find *de novo* the OTU representatives using the UPARSE algorithm. This step also removes *de novo* chimeric reads, resulting in a set of OTU representatives of very high sequence quality [33].

### Taxonomy Assignment

Out of many possible ways of assigning taxonomy for pared-end 16S reads, we have decided to use k-mer-based methods, since, besides their speed and accuracy, they allow us to query reads with gaps in them (marked as unknown nucleotides) without loss of accuracy. In particular, we use mothur's implementation [9] of the Ribosomal Database Project's naive Bayesian classifier [34]. For this step, we classify the reads against the Greengenes 13_–_5 database, or in the case of the paired reads, we look up the OTU representatives in the read library built for this purpose in the “Merging of Reads, Length Trimming and Concatenation” section and classify those reads. After classification, we remove fully unclassified reads from the OTU representatives, as they are presumed contaminants (in most cases, these are phiX reads leftover from sequencing).

### Structural Alignment

After taxonomy assignment, reads R1 and R2 are aligned using Infernal version 1.1 [28], using a secondary structure model created using the Ribosomal Database Project's (RDP) [35] recommended training set. As per RDP recommendations, options for the aligner are set at “-g –sub –notrunc” (with the “–g” and “–notrunc” options added as a requirement in version 1.1 of Infernal). Structural alignments produces better multiple sequence alignments of 16S rDNA short reads amplicons (see Fig. 1) than other template-based methods [30]. For the paired reads, the reads are split into their R1 and R2 components by looking up the corresponding reads in the R1 and R2 files, and then aligned separately using Infernal. This is necessary because Infernal does not expect a large gap in between the two components, otherwise alignment will fail. After alignment, the scores are analyzed and all alignments with negative scores are removed, as well as the corresponding unaligned reads. These negative scores indicate reads that aligned very poorly to the secondary structure model. For the paired alignments, after removing these low scoring reads, the R1 and R2 components are concatenated back together.

### Mapping to OTU Representatives

The reads output in the “Merging of Reads, Length Trimming and Concatenation” section are then mapped against their corresponding set of unaligned, clean OTU representatives using USEARCH at 97% sequence identity. The resulting table is then parsed to obtain the different OTU counts per sample, and finally combined with taxonomy information and other metadata into a BIOM-formatted [36] file using the biom-format python package. The unmapped reads are presumed to be either chimeric reads, contaminants or singletons distant to any reported OTU.

### Generation of Phylogenetic Trees

In order to obtain the evolutionary relationships between the members of the microbial communities, the clean alignments obtained in the “Structural Alignment” section are used to create a phylogeny. Empty columns are removed from the alignments of the OTU representatives, and the trees are calculated using FastTree version 2.1.7 [29], using options “-nt -gtr -gamma.”

## Methods for Validation Using Synthetic Communities

To asses the performance and accuracy of our choice of primers and tools when compared to full-length 16S reads, we used 100 synthetic communities of 20 samples each, for two pairs of primers targeting regions V3–V5 and V6–V9. We ran a simplified version of our pipeline and compared *β*-diversity metrics and phylogeny metrics to quantify differences with the equivalent result obtained from the corresponding full-length 16S reads.

### Input Data

To create our synthetic communities, we started with the complete 16S Greengenes 13_–_5 database. Using the PrimerProspector tool [37] we selected artificial paired amplicons matching forward primer 357F (CCTACGGGAGGCAGCAG) and reverse primer 926R (CCGTCAATTCMTTTRAGT) for the V3-V5 primer pair, each of length 250 bp. For the V6-V9 primer pair, we used forward primer 968F (AACGCGAAGAACCTTAC) and reverse primer 1492R (CGGTTACCTTGTTACGACTT), to obtain amplicons also of length 250 bp. For the full length reads, we selected artificial amplicons matching primers 27F (AGAGTTTGATCMTGGCTCAG) and 1492R (CGGTTACCTTGTTACGACTT). We kept reads whose IDs were found both in the paired-end and full-length libraries, as well as having no ambiguous nucleotide characters in their reads. The total number of reads surviving this selection was 142,610 reads for the V3–V5 library, and 143,085 reads for the V6–V9 library. No artificial quality scores were created, hence the reads will be assumed to be perfect.

### Synthetic Communities

For each of the primer pairs used for validation, we created 100 synthetic communities, each with 20 samples. Each sample has 2,500 unique IDs randomly picked from the selected read set from the “Input Data” section. For each of the IDs we created a set of clone reads whose size was chosen from a random distribution resembling a log-series model of ecological communities [38]. The selected IDs with their respective multiplicities were used to select the corresponding reads from the master full-length and paired-end libraries to create three different sets of communities with identical IDs: full-length, R1 and R2.

### Merging of Reads and Concatenation for Validation

As in the “Merging of Reads, Length Trimming and Concatenation” section. the R1 and R2 libraries were grouped into a single file for each read. Read lengths in both library R1 and R2 were left unmodified. A third library was created by concatenating the corresponding reads from both R1 and R2, created the paired library.

### Finding OTU Representatives for Validation

The three libraries from the “Merging of Reads and Concatenation for Validation” section and the full-length library are dereplicated, building clusters of reads with 100% similarity using mothur, and annotated with cluster size. To ensure the use of high quality reads when finding the OTU representatives, singleton reads are discarded. No inspection of read lengths is necessary because, in the case of libraries R1, R2 and paired, the reads are of uniform length by construction, and in the case of the full-length library, the reads are globally alignable by construction (from primers at both ends of the reads). Reads are sorted by cluster size and processed using USEARCH to find *de novo* the OTU representatives using the UPARSE algorithm. This step also removes *de novo* putative chimeric reads, resulting in a set of OTU representatives of very high sequence quality [33].

### Structural Alignment for Validation

Libraries R1, R2 and full-length are aligned using Infernal version 1.1 [28], using a secondary structure model created using the Ribosomal Database Project's (RDP) [35] recommended training set. As per RDP recommendations, options for the aligner are set at “-g –sub –notrunc” (with the “–g” and “–notrunc” options added as a requirement in version 1.1 of Infernal). For the paired reads, the reads are split into their R1 and R2 components by looking up the corresponding reads in the R1 and R2 libraries, and then aligned separately using Infernal. This is necessary because Infernal does not expect a large gap in between the two components, otherwise alignment will fail. Alignment scores are not inspected since the reads are free of contaminants by construction.

### Mapping to OTU Representatives for Validation

The reads output in the “Merging of Reads and Concatenation for Validation” section are then mapped against their corresponding set of unaligned, clean OTU representatives using USEARCH at 97% sequence identity. The resulting table is then parsed to obtain the different OTU counts per sample, and finally combined with taxonomy information and other metadata into a BIOM-formatted [36] file using the biom-format python package. The unmapped reads are presumed to be either chimeric reads or singletons distant to any reported OTU.

### Generation of Phylogenetic Trees for Validation

In order to obtain the evolutionary relationships between the members of the microbial communities, the clean alignments obtained in the “Structural Alignment for Validation” section are used to create a phylogeny. Empty columns are removed from the alignments of the OTU representatives, and the trees are calculated using FastTree [29]. These trees will be used for comparisons of *β*-diversity.

These trees are not suitable for direct comparison of the phylogenetic structure between the different libraries, since the OTU picking procedure may choose different reads as representatives, as well as a different number of OTUs. To solve this, we took as a reference the trees created for the full-length reads, created the equivalent libraries with R1, R2 and paired reads and re-run the alignment and calculate the new trees. This way, the trees will have the same number of leaves, same leaf IDs and hence directly comparable.

### Comparison of *β*-Diversity Between the Libraries for Validation

For each of the 100 synthetic communities, we took the resulting BIOM files and phylogenetic trees (for all four libraries, R1, R2, paired and full-length), and calculated the unweighted UniFrac distance matrix [31] between the 20 samples of each community, using QIIME 1.7.0 [8]. We compared the distance matrices of the libraries R1, R2 and paired to the corresponding full-length distance matrix using a Mantel correlation test, as implemented by the R package vegan [39] (see Fig. 3), and assessed the significance of the results using a signed, ranked Wilcoxon test as implemented in R.

### Comparison of Phylogenies Between the Different Libraries for Validation

For each of the 100 synthetic communities, we took the full-length tree and the equivalent and comparable trees created using libraries R1, R2 and paired, and calculated the cophenetic distance matrix (which measures the distances between leaves of the phylogenetic tree through branch lengths), as implemented in the R package ape [40]. The distance matrices were compared using a Mantel correlation test, as implemented in the R package vegan [39] (see Fig. 2), and assessed the significance of the results using a signed, ranked Wilcoxon test as implemented in R.

### Data Availability

All the data created for the synthetic communities analysis, including reads, resulting OTU tables and scripts, are available for download from the Dryad repository. The data DOI is doi: 10.5061/dryad.fm67n.

## Methods for Validation Using a Sample-Modeled Synthetic Mock Community

As another validation of our approach, we used an existing set of read libraries from stool samples to create a synthetic mock community based on real gastrointestinal microbiome data, for a more realistic case testing.

### Ethics Statement and Sample Collection

Subjects were consented under IRB #11-002697, which was approved and reviewed by the Mayo Clinic Institutional Review Board. Written consent was provided by all subjects. A total of 20 multiple sclerosis patients were consented under Mayo Clinic IRB #11-002697 and stool samples were collected either in clinic or using a specimen mailer tube. Upon arrival, samples were frozen at −80°C until they were ready to be processed.

### DNA Library Preparation

DNA extraction was carried out according to the Manual of Procedures on the Human Microbiome Project website http://www.hmpdacc.org using physical and chemical lysis with a FastPrep-24 (MP Biomedicals, Santa Ana, CA) and PowerSoil Extraction Kit (MoBio, Carlsbad, CA). Amplification targeted V3 and V5 regions, whose performance has been previously characterized [41], [42], using primers 357F (AATGATACGGCGACCACCGAGATCTACACTATGGTAATTGTCCTACGGGAGGCAGCAG) and 926R (CAAGCAGAAGACGGCATACGAGAT-NNNNNNNNNNNN-AGTCAGTCAGCCCCGTCAATTCMTTTRAGT) with barcodes 55–76 from Caporaso *et al.* [10]. PCR was run through 34 cycles of 98°C for 15 seconds, 70°C for 20 seconds, 72°C for 15 seconds, in accordance to the temperatures from the polymerase hotstart mix for Kapa Hi-Fi (Kapa Biosystems, Boston, MA). Electrophoresis was then carried out using a 2200 TapeStation (Agilent Technologies, Santa Clara, CA). After verifying there were no other large bands besides the amplicon, purification was carried out using magnetic beads. Concentrations for each sample were then diluted to 10 nM and pooled for sequencing. Amplicons were then sequenced on a MiSeq sequencer (Illumina, San Diego, CA) using a 500 cycle kit and custom read1 (TATGGTAATTGTCCTACGGGAGGCAGCAG), read2 (AGTCAGTCAGCCCCGTCAATTCMTTTRAGT), and index (ACTYAAAKGAATTGACGGGGCTGACTGACT) sequencing primers.

### Sample Modeling

To create the synthetic full-length 16S samples, we mapped five of the short-read libraries onto the full Greengenes 13_–_5 database using USEARCH's “-usearch_–_global” option [43], selecting the database reads that matched at 97% sequence identity. Using PrimerProspector [37] we extracted 250 bp long V3 and V5 paired reads from the matches, and also created an almost-full-length 16S library by trimming their read lengths down to 1300 bp long.

### OTU Representatives, Alignment and Phylogeny

As in the previous synthetic library processing, we dereplicated each library, sorted by cluster size, discarded singleton clusters and picked OTUs at 97% sequence identity using USEARCH [33]. Then the libraries were mapped onto the OTU representatives using USEARCH to obtain the OTU sizes. We multiple sequence aligned the OTU representatives to a model secondary structure of the 16S gene using Infernal 1.1 [28] and created a phylogenetic tree using FastTree 2.1.7 [29]. All of the above information was consolidated into BIOM files using IM-TORNADO scripts.

### Comparison of *β*-Diveristy

For each of the BIOM files correspoonding to each library (R1, R2, paired and full-length), we calculated the corresponding unweighted UniFrac distance matrix using QIIME 1.7.0 [8]. We compared the matrices of the three short libraries (R1, R2 and paired) against the full-length UniFrac matrix using a Mantel correlation test, as implemented in the R package vegan [39].

### Data Availiability

All the data created for the sample synthetic mock community, including original reads and synthetic reads, resulting OTU tables and scripts, are available for download at the Dryad repository. The data DOI is doi: 10.5061/dryad.fm67n.

## Discussion

We have shown that the use of paired reads improves performance over R1 or R2 alone. Moreover, significance tests revealed that in all cases R1 and R2 together almost always provided a better reflection of the results from full length 16S rDNA. By itself, this fact is unsurprising since R1 or R2 both use only a subset of the information available to paired reads, which are made up of both R1 and R2 together. On the other hand, what may seem more surprising is that paired read approaches are not utilized as the norm. However, the overall gains from using paired reads is moderate when considering the enormous amount of book-keeping and tracking involved (shown in Fig. 4). It is clear that the travails of making maximal use of paired reads outweighs the benefit for many microbiome researchers. It is our hope that with the introduction and public availability of IM-TORNADO that microbiome researchers will be able to overcome the logistical barrier to use of non-overlapping paired-end 16S rDNA reads.

It is our view that the most valuable tools are also the most widely usable. Now more than ever, community standards within the field of microbiome research are becoming increasingly important as the field (and the data) continues to grow at a rapid pace. One of the important design principles of IM-TORNADO is our adherence to already widespread analyses in microbiome studies. This allows results from IM-TORNADO to be readily compared to results from pipelines such as QIIME or mothur [8], [9]. Furthermore, we utilize the BIOM format popularized by QIIME which allows for our results to be manipulated by existing tools that accept the BIOM format, such as mothur and phyloseq [44]. The scripts and tools for running IM-TORNADO are available at http://sourceforge.net/projects/imtornado.

Finally, as an important consideration in future studies, the ability to use non-overlapping paired end 16S rDNA reads means added flexibility to experimental design. This comes in direct contrast to overlapping paired read designs which require primers to be adjacent to each other. While overlapping primer design has potential advantages (see Supplemental Materials) and could in principle use nearly the same amount of information as non-overlapping reads, with a small number of bases given to assemble the reads, primer choice ideally would vary between studies due to emphasis on different taxa in particular environments. Creating a means for the more holistic analysis of non-overlapping paired reads allows for greater flexibility in primer choice, allowing researchers to target a larger variety of regions to maximize sensitivity and specificity for a particular environment without using only adjacent regions or resorting to single end read analysis. This applies not only to 16S applications. For example, studies using the 18S rDNA gene [45] will benefit from a paired-end sequencing approach. And these benefits extend to any other structurally alignable RNA molecule. Being able to utilize non-overlapping reads also ameliorates issues arising from poor sequencing quality and shorter than expected read lengths after quality trimming by enabling analysis of data from lower quality sequencing runs.

## Conclusions

We implemented a workflow for computing taxonomy, OTUs, and phylogeny from non-overlapping 16S rDNA paired-end reads. As a quality metric for our particular implementation, we demonstrate the differences between the use of single versus non-overlapping paired-end reads. This is meant to address another possible methodology for data analysis in studies that utilize primer designs where there are non-overlapping paired-end reads [12], [15].

As shown by the results of our tests on synthetic mock datasets in Tables 1 and 2, as well as Figs. 2 and 3, including both reads in the computational analysis of taxonomic classification, phylogeny, and *β*-diversity produces results that have greater agreement with those from full length 16S rDNA then when using either single-end read alone. Given the implied increase in accuracy when analyzing environmental data, it is evident that paired-end analysis should be used when possible in these cases.

## Supporting Information

S1 Table **Taxonomy comparison.** Accuracy of taxonomy assignments for different read lengths, with errors and read trimming. The table shows the effect of base errors in the accuracy of the taxonomy assignment process (Paired, with errors, average 15.6 errors per read pair, standard deviation 3.7). It also shows the improvement in accuracy after applying the read trimming step pf the pipeline (Paired, trimmed, with errors, average 6.0 errors per read pair, standard deviation 2.9, average read pair length 438.7 bp, standard deviation 28 bp).
(PDF)

S2 Table **Comparison of OTU counts for synthetic mock communities.** Comparison of OTU counts at 97% sequence identity, averaged for the 100 synthetic mock communities used for validation. From the table we can observe the trend of Full length reads showing comparable or higher OTU counts than the short read types, and among these short reads, R1 shows the higher OTU counts.
(PDF)

S1 Text **Supplemental Information for “IM-TORNADO: A Tool for Comparison of 16S Reads from Paired-End Libraries”.**
(PDF)

S1 Code **Source code for the IM-TORNADO pipeline, version 2.0.0.** This and subsequent versions of the pipeline are available in the SourceForge repository at http://sourceforge.net/projects/imtornado.
(GZ) Click here for additional data file.
