# Supplementary material for: IM-TORNADO: A Tool for Comparison of 16S Reads from Paired-End Libraries
Source: PLoS One. 2014 Dec 15;9(12):e114804. doi: 10.1371/journal.pone.0114804 (PMC4266640; doi:10.1371/journal.pone.0114804)
Supplement: S1 Table — Taxonomy comparison. Accuracy of taxonomy assignments for different read lengths, with errors and read trimming. The table shows the effect of base errors in the accuracy of the taxonomy assignment process (Paired, with errors, average 15.6 errors per read pair, standard deviation 3.7). It also shows the improvement in accuracy after applying the read trimming step pf the pipeline (Paired, trimmed, with errors, average 6.0 errors per read pair, standard deviation 2.9, average read pair length 438.7 bp, standard deviation 28 bp). (PDF) [file pone.0114804.s001.pdf]

Library type	Domain	Phylum	Class	Order	Family	Genus	Species
Paired (error-free, 2x250 bp)	100.0	99.95	99.91	99.59	98.25	94.32	90.78
Paired (error-free, 2x200 bp)	100.0	99.95	99.90	99.52	97.84	93.47	89.14
Paired (error-free, 2x150 bp)	100.0	99.94	99.87	99.41	97.61	92.59	88.15
Paired (error-free, 2x100 bp)	100.0	99.91	99.80	99.11	96.85	90.43	83.75
Paired (with errors, 2x250 bp)	100.0	99.84	99.67	98.26	96.19	88.48	80.25
Paired (trimmed, with errors, 2x250 bp)	100.0	99.91	99.84	99.14	97.71	92.73	87.73
