# Supplementary material for: IM-TORNADO: A Tool for Comparison of 16S Reads from Paired-End Libraries
Source: PLoS One. 2014 Dec 15;9(12):e114804. doi: 10.1371/journal.pone.0114804 (PMC4266640; doi:10.1371/journal.pone.0114804)
Supplement: S2 Table — Comparison of OTU counts for synthetic mock communities. Comparison of OTU counts at 97% sequence identity, averaged for the 100 synthetic mock communities used for validation. From the table we can observe the trend of Full length reads showing comparable or higher OTU counts than the short read types, and among these short reads, R1 shows the higher OTU counts. (PDF) [file pone.0114804.s002.pdf]

Library type	V3-V5 OTU counts (mean ± std. dev.)	V6-V9 OTU counts (mean ± std. dev.)
Paired	3172.0 ± 39.9	3245.0 ± 38.2
R1	3589.4 ± 42.5	3283.8 ± 32.2
R2	2782.4 ± 35.9	3113.5 ± 34.5
Full length	3567.7 ± 42.5	3483.8 ± 38.3
