# Supplementary material for: IM-TORNADO: A Tool for Comparison of 16S Reads from Paired-End Libraries
Source: PLoS One. 2014 Dec 15;9(12):e114804. doi: 10.1371/journal.pone.0114804 (PMC4266640; doi:10.1371/journal.pone.0114804)
Supplement: S1 Text — Supplemental Information for “IM-TORNADO: A Tool for Comparison of 16S Reads from Paired-End Libraries”. (PDF) [file pone.0114804.s003.pdf]

Supplemental Information for "IM-TORNADO: A Tool for Comparison of 16S Reads from Paired-End Libraries"

Patricio Jeraldo[1,2], Krishna Kalari[3], Xianfeng Chen[3], Jaysheel Bhavsar[3], Ashutosh Mangalam[4], Bryan White[2,5], Heidi Nelson[1], Jean-Pierre Kocher[3], Nicholas Chia[1,2,6,*]

1 Department of Surgery, Mayo Clinic, Rochester, MN, USA

2 Institute for Genomic Biology, University of Illinois at Urbana-Champaign, Urbana, IL, USA

3 Department of Health Sciences Research, Mayo Clinic, Rochester, MN, USA

4 Department of Immunology, Mayo Clinic, Rochester, MN, USA

5 Department of Animal Sciences, University of Illinois at Urbana-Champaign, Urbana, IL, USA

6 Department of Physiology and Biomedical Engineering, Mayo Clinic College of Medicine, Rochester, MN, USA

1 Accuracy of taxonomy assignments in the presence of sequencing errors

Although the IM-TORNADO pipeline focuses on the use of non-overlapping reads, there are valuable questions to be explored in the comparison of non-overlapping primer designs versus overlapping designs. As an aside, we here address some of these issues. While this does not help in the case of many of the already existing datasets that have already been sequenced, especially with shorter read length technologies where overlap is difficult to achieve, it does inform future experimental designs.

The main question that arises between non-overlapping versus overlapping reads is the issue of length versus quality. In the case of non-overlapping reads, one utilizes the quality scores in order to trim each read to a length where the error rates remain acceptable. In contrast, overlapping reads provide a means of error correction at the cost of sequencing some region twice. This has the advantage of being able to better assess the real error rates rather than rely on the quality scores alone by looking for discrepancies between read 1 and read 2.

In this test, our non-overlapping reads are subjected to mutations according to realistic error profiles. These are tested with and without quality trimming for comparison purposes. Realistically, these would always be trimmed when analyzed. We simulate the results from overlapping primer design by assuming

that overlapping reads provide perfect sequences. While this may be a bit exaggerated, it nonetheless provides an important bound on the performance of overlapping reads and seems somewhat justifiable given the multiplicative nature of the fidelity rate when there is overlap. The main sacrifice made for these lower error rates is the loss of total sequence length since one has to, in effect, sequence the same positions twice in order to achieve them. Therefore, we tested perfect reads at 4 different lengths, 250, 200, 150, and 100 bases for each read (or 500, 400, 300, and 200 bases of total amplicon length). We kept the V3-V5 design present in the taxonomy test shown in the main article. While this means that the reads we used are not in fact overlapping, the test design is meant to simulate the effect that overlap would have on the error rates. Also, the different lengths examined are meant to mimic the effect of sequencing some regions twice, which means necessarily shortening the effective length of the amplicon region used.

Results

As shown in Table S1, paired reads with errors included show a marked decrease in accuracy, when compared to the equivalent 2x250 bp error-free reads, specially at Genus and Species level assignments. When trimming the reads to remove low quality bases using Trimmomatic as used in the IM-TORNADO pipeline, the taxonomic assignment accuracy improves, despite the decrease in length of the reads 60 bases on average.

When comparing the assignment accuracy trimmed reads with error-free paired reads with different read lengths, the trimmed reads perform as similarly as reads between 300 bp long (2x150 bp) and 400 bp long (2x200 bp). In short, this 2x250 bp construct with no overlaps (including trimming of most errors) performs comparably to construct of 400 base pairs with perfect reads, validating the quality of the resulting data. Longer read constructs, such as 2x300 bp, can only improve the taxonomic assignment quality.

Methods

Generation of simulated paired-end reads

Simulated paired-end reads were generated using the ART [1] next-generation read simulator, in amplicon mode. The source amplicons were constructed from the Greengenes 13_5 database [2] using the PrimerProspector tool [3] with primers for the v3-v5 region of 16S rDNA, namely 357F (CCTACGGGAGGCAGCAG) and 926R (CCGTCAATTCMTTTRAGT). 100 replicate libraries of 250 bp long paired-end reads were created using an appropriate error model for ART (see Supplemental files), which is used to model PHRED quality score and error probability. For each library created using ART, a SAM file is created with information about the location of the errors, encoded as a CIGAR string, and the simulated PHRED score. Also, error free libraries were created for lengths of 100 bp, 150 bp and 200 bp.

Read trimming and error counting

Trimming of reads to remove low-quality ends was performed using Trimmomatic [4] using the same parameters as the pipeline, namely "LEADING:3 TRAILING:3 SLIDINGWINDOW:4:15." Counting of the per-read errors for both trimmed and untrimmed reads was performed by parsing the corresponding CIGAR string using a custom script (see Supplemental files), accounting for the length of the trimmed read.

Taxonomy assignment

For each of the trimmed, untrimmed and error-free libraries, we assigned taxonomy to the reads using the Ribosomal Database Project's naïve Bayesian Classifier [5] as implemented in mothur [6]. Resulting taxonomy profiles were compared to the actual taxonomy assignment for each read based on the read IDs (which are the Greengenes IDs of the source, full-length 16S reads). Comparison was performed at each of the canonical taxonomy levels, namely Domain, Phylum, Class, Order, Family and Species.

Data availability

Data for this validation test, including synthetic mock libraries, source sequences, taxonomic assignments, scripts and result tables, as well as data for all validation tests described in the main article, is available at the Dryad repository. The data DOI is doi:10.5061/dryad.fm67n.

Discussion

In summary, our test indicates that an overlap design that produces an amplicon of 400 bases will perform as well as a non-overlapping design that is sequenced using a 500 cycle kit.

2 OTU counts

While the question of clustering and operational taxonomic units (OTUs) is interesting, it is one that becomes confounded by multiple factors that arise from differences in evolutionary rates across 16S rDNA. Nonetheless, it can be an illuminating exercise and therefore we have provided Table S2, which shows the different number of OTUs derived from R1, R2, and paired analyses of our V3-V5 and V6-V9 synthetic mock datasets used in the main article. Overall, the number of OTUs produced by R1 and paired analyses is comparable to the number of OTUs detected using full length 16S rDNA sequences.

References

1. Huang W, Li L, Myers JR, Marth GT (2012) ART: a next-generation sequencing read simulator. Bioinformatics 28: 593–594.

2. DeSantis TZ, Hugenholtz P, Larsen N, Rojas M, Brodie EL, et al. (2006) Greengenes, a Chimera-Checked 16S rRNA Gene Database and Workbench Compatible with ARB. Appl Environ Microbiol 72: 5069–5072.

3. Walters WA, Caporaso JG, Lauber CL, Berg-Lyons D, Fierer N, et al. (2011) PrimerProspector: de novo design and taxonomic analysis of barcoded polymerase chain reaction primers. Bioinformatics 27: 1159–1161.

4. Bolger AM, Lohse M, Usadel B (2014) Trimmomatic: a flexible trimmer for Illumina sequence data. Bioinformatics 30: 2114–2120.

5. Wang Q, Garrity GM, Tiedje JM, Cole JR (2007) Naive Bayesian classifier for rapid assignment of rRNA sequences into the new bacterial taxonomy. Appl Environ Microbiol 73: 5261–5267.

6. Schloss PD, Westcott SL, Ryabin T, Hall JR, Hartmann M, et al. (2009) Introducing mothur: open-source, platform-independent, community-supported software for describing and comparing microbial communities. Appl Environ Microbiol 75: 7537–7541.
